# Mutual interaction of microbiota and host immunity during health and diseases

**DOI:** 10.52601/bpr.2021.200045

**Published:** 2021-08-31

**Authors:** Di Wu, Yinlian Zhang, Suwei Dong, Chao Zhong

**Affiliations:** 1 Institute of Systems Biomedicine, Department of Immunology, Beijing Key Laboratory of Tumor Systems Biology, Peking University Health Science Center, Beijing 100191, China; 2 State Key Laboratory of Natural and Biomimetic Drugs, Department of Chemical Biology, School of Pharmaceutical Sciences, Peking University, Beijing 100191, China

**Keywords:** Microbiota, Microbial-derived antigens and metabolites, Immune system, Composition of microbiota, Host health and diseases

## Abstract

Microbiota–host interaction has attracted more and more attentions in recent years. The association between microbiota and host health is largely attributed to its influence on host immune system. Microbial-derived antigens and metabolites play a critical role in shaping the host immune system, including regulating its development, activation, and function. However, during various diseases the microbiota–host communication is frequently found to be disordered. In particular, gut microbiota dysbiosis associated with or led to the occurrence and progression of infectious diseases, autoimmune diseases, metabolic diseases, and neurological diseases. Pathogenic microbes and their metabolites disturb the protective function of immune system, and lead to disordered immune responses that usually correlate with disease exacerbation. In the other hand, the immune system also regulates microbiota composition to keep host homeostasis. Here, we will discuss the current advances of our knowledge about the interactions between microbiota and host immune system during health and diseases.

## INTRODUCTION

Microbiome refers to bacteria, fungi, and virus that colonize in our body. During the past few years, our knowledge about gut microbiota, especially the bacteria component, has developed largely. The estimated bacteria colonized in the gut can reach to 100 trillion, more than 10 times of the human cell number. Moreover, metagenome of the gut microbiota is even more abundant. These complexities account for the crucial but also complicated role of gut microbiota during health and diseases.

Growing evidence suggests that the hosts are affected by gut microbiota largely through its impact on immune system. In supporting, germ-free (GF) mice which lack the microbiota exhibit a dramatic deficiency of immune function, especially in the gut (Gensollen *et al*. 2016). Mucosal surface and skin are interfaces for the microbiota–immune system interplay. After birth, the development of immune system and microbiota colonization concurrently occurs, enabling a close interaction between them (Belkaid *et al*. 2017). Mechanically, microbial-derived antigens or active metabolites usually play critical roles in regulating the development, activation and function of immune system.

In this review, we will summarize the current knowledge about microbiota and host immune system interaction. In addition, occurrence or progression of many diseases are frequently associated with disordered microbiota and immune responses. So, we will further discuss how the disrupted microbiota–host interaction is correlated with diseases. In particular, we highlight the following aspects: (1) the role of microbiota as well as microbial-derived antigens and metabolites in shaping the innate and adaptive immune systems; (2) the correlation of disordered microbiota–immune system interaction with various diseases; (3) the impact of host immunity on microbiota composition and tissue homeostasis.

## MICROBIOTA FACILITATES THE SHAPING OF IMMUNE SYSTEM

### Immune system development

Microbiota–immune system interaction happens as early as during delivery in genital tract. After then, the infant body is quickly colonized by microbiota. The composition of microbiota in various tissues experience dynamic changes, associated with the physiological and environmental changes during growth. Meanwhile, the postnatal period is also critical for immune system development. As reported, microbial-derived antigens and metabolites play essential roles in regulating the development of immune system such as formation of lymphoid structures and education of immune cells (Gensollen *et al*. 2016).

#### Lymphoid structure formation

Gut-associated lymphoid tissues (GALTs) form the largest immune network in our body. They are essential in maintaining gut homeostasis. GALTs include secondary lymphoid organs such as mesenteric lymph nodes (mLNs) and Peyer’s patches (PPs), as well as tertiary lymphoid organs such as cryptopatches (CPs) and isolated lymphoid follicles (ILFs). Gut microbiota plays a critical role in the development and maturation of GALTs. Besides, it also regulates the development of other mucosa-associated lymphoid tissues (MALTs) and peripheral lymphoid organs. The germ-free mice exhibited an obvious deficiency of immune system, including decreased immune cells, disorganized structure of secondary lymphoid organs, and reduction and immaturation of tertiary lymphoid structures. Microbiota transplantation could significantly recover the development and maturation of GALTs. Furthermore, a recent study suggested that a specific bacterial polysaccharide (PSA) from *Bacteroides fragilis* could also efficiently restore the number of CD4^+^ T cells, indicating that microbial metabolites played a crucial role in promoting the development and maturation of immune system (Oerlemans *et al*. 2021). The formation of secondary and tertiary lymphoid organs needs a particular type of immune cells, termed lymphoid tissue inducer (LTi). Lymphotoxin-β generated by these cells plays an essential role in lymphoid organogenesis (Upadhyay *et al*. 2014). The LTi activation is regulated by upstream cytokines, mainly including IL-23 and IL-1β. They are secreted by dendritic cells, macrophages, and gut epithelial cells under the stimulation of microbiome. The lymphotoxin signal induces an inflammatory environment in local tissue and promotes chemokine production. It leads to lymphocyte recruitment to tertiary lymphoid organs to promote the structure maturation (Upadhyay *et al*. 2014). Once matured, these lymphoid structures are more prominent in promoting tissue homeostasis and regulating immune responses. Therefore, microbiota is critical for the development and maturation of lymphoid structures.

#### T cell development

T cell development happens in thymus. Self-antigen expression on thymic epithelial cells (TECs) is important in generating the TCR repertoire and central tolerance. Thus, microbial antigens were thought to be absent in T cell development. However, a recent study revealed that intestinal microbiota colonization in early life could induce the expansion of microbiota-specific T cells in thymus (Zegarra-Ruiz *et al*. 2021). The intestinal dendritic cells migrated from gut to thymus, and they delivered microbial antigens for the generation of microbial antigen specific T cells. After development, these microbial antigen specific T cells migrated to the gut. There they encounter the microbial-derived antigens again and further differentiated to effector cells. This process was critical in regulating the gut homeostasis.

### Innate immunity

The communication between gut microbiota and host is largely relayed on innate immune system. As well known, dendritic cells and macrophages in the gut epithelium can sense pathogen-associated molecular patterns (PAMPs) on the microbiome. Then, they are activated and transduce the gut microbial signals to other immune cells. In steady state, their activation leads to an immune tolerance to gut commensals or a resistance to pathogenic microbes. In this part, we will also introduce some other recently studied innate immune cells.

#### Dendritic cells

The Dendritic cells (DCs) population consists of classical DC (cDC), plasmacytoid DC (pDC) and monocyte-derived DC (Sun *et al*. 2020). They play a critical role in maintaining tolerance to gut commensals and resistance against pathogenic microbes. Gut homing DCs can process microbial antigens and present their peptides on MHC molecules to induce activation of microbial specific adaptive immune cells. Microbial derived metabolites also affect the DC function. Retinoic acid (RA) is a vitamin A metabolite. It plays an important role in gut-tropic pre-mucosal dendritic cell (pre-uDC) generation. It upregulates integrin α_4_β_7_ expression on the pre-uDC, which benefits their homing toward intestine (Zeng *et al*. 2013). In addition, RA promotes differentiation of pre-uDC into cDC1 and cDC2. Deficiency of RA will result in a reduction of cDC2, as well as phenotype changes of cDC1 and cDC2 (Zeng *et al*. 2016). DCs were also reported to be regulated by vitamin D. DCs treated with 1,25(OH)_2_D_3_, the active form of vitamin D, or vitamin D analogs showed resistance to maturation under inflammatory stimuli (Aranow 2011). 1,25(OH)_2_D_3_ inhibited the expression of MHC-II and costimulatory molecules on DCs. Moreover, 1,25(OH)_2_D_3_ promoted DCs to secret anti-inflammatory cytokine IL-10, which induced Treg differentiation (Aranow 2011; Martens *et al*. 2020). Short-chain fatty acids (SCFAs) refer to fatty acids with less than six carbons, predominantly including acetic acid, propionic acid, and butyric acid. They are generated through the bacterial fermentation of dietary fibers. SCFA is another critical microbial metabolite for DCs. Acetate, butyrate, and propionate could inhibit the expression of costimulatory molecules (CD80, CD86 and CD40) on DCs. They also repressed the production of several pro-inflammatory chemokines and cytokines (Iraporda *et al*. 2015; Nastasi *et al*. 2015). SCFAs treated DC displayed a strong Treg-inducing activity, promoting Foxp3 expression in naïve CD4^+^ T cells (Arpaia *et al*. 2013). Therefore, the microbiota and microbial metabolites are necessary to regulate the maturation and function of DCs.

#### Macrophages

Macrophages play important roles in maintaining tissue homeostasis. They recognize foreign pathogens by pattern recognition molecules such as TLR4, and then are activated to eliminate the invading pathogens (Swanson *et al*. 2020). Under distinct stimuli, macrophages are polarized to classical (“M1”) or alternative (“M2”) activated subtypes. They play pro- or anti-inflammatory roles respectively. Microbiota-derived metabolites also affect the macrophages function. For example, lipopolysaccharides (LPS) from gram-negative bacteria could be recognized by TLR4 on macrophages and promoted them to produce inflammatory cytokines (Correa-Oliveira *et al*. 2016; Fujihara *et al*. 2003). The LPS also converted macrophages from an M2 phenotype to the M1 phenotype. In opposite, short chain fatty acid facilitated M2 polarization. Propionic acid is normally generated by the gut flora in colon. They inhibited the production of pro-inflammatory cytokines and chemokines from macrophages (Al-Lahham *et a*l. 2012). Another SCFA butyrate was reported to suppress LPS-mediated macrophages migration (Maa *et al*. 2010). Particularly in the gut, macrophages are divided into a “resident” and an “inflammatory” subset (Bain *et al*. 2013). The resident macrophages secret anti-inflammatory cytokine IL-10 and thus promote Treg cell expansion (Isidro *et al*. 2016). On the other hand, the inflammatory macrophages express high levels of CD14 and secret pro-inflammatory cytokines (Bain *et al*. 2013). The gut microbiota-generated butyrate suppressed the inflammatory macrophages through inhibiting histone deacetylation or NF-κB activation (Chang *et al*. 2014). In addition, it also facilitated anti-inflammatory activity of macrophages by promoting IL-10 secretion (Singh *et al*. 2014). Vitamin D also regulates the macrophage function. 1,25(OH)_2_D_3_, increased the antimicrobial activity of macrophages by promoting the production of defensin 2 and cathelicidin antimicrobial peptide (CAMP) (Bellan *et al*. 2020). In brief, the microbiota and microbial metabolites play important roles in regulating the migration and function of macrophages.

#### Innate lymphoid cells

As mentioned above, the lymphoid organogenesis is regulated by LTi, which are classified into an innate lymphoid cell (ILC) population. The ILC is a recently defined component of the innate immune system. ILCs play an essential role in regulating tissue homeostasis. According to their functional features, the ILCs are divided into three subgroups. The group 1 ILC (ILC1) is prominent in secreting IFN-γ and TNF-α. The group 2 ILC (ILC2) mainly expresses IL-5, IL-13, and certain IL-4. And, the group 3 ILC (ILC3), including a NCR^+^ ILC3 subset and a CCR6^+^ LTi subset, are prominent in generating IL-22 and IL-17A (Spits *et al*. 2013; Vivier *et al*. 2018).

Microbiota is critical in regulating ILCs. In germ-free mice, the activity of NK cells, which belong to ILC1, was significantly reduced in non-mucosal tissues. This led to a severe defect in their antiviral immunity. Further investigation revealed that phagocytes in the germ-free mice failed to express several inflammatory response-related genes that were essential to prime NK cells (Ganal *et al*. 2012). The lack of microbial colonization in germ-free mice also impaired NCR^+^ ILC3 development, and reduced their production of IL-22 (Negi *et al*. 2019). Transferring microbiota of SPF mice to the germ-free mice efficiently recovered the expression of IL-22 from ILC3, confirming that microbiota regulated ILC3 activation (Reynders *et al*. 2011). Mechanically, microbiome colonization promotes IL-23 production in the gut which was essential for the ILC3 function. Besides, segmented filamentous bacteria (SFB), a particular commensal, was sufficient to promote IL-22 production from ILC3 (Sano *et al*. 2015).

An important manner that microbiota affects the development and function of ILCs is through microbial-derived metabolites. For instance, SCFAs could bind to “metabolite-sensitive” G protein-coupled receptors (GPCRs) on immune cells and transduce regulatory signals to them (Koh *et al*. 2016; Tan *et al*. 2017). The NCR^+^ ILC3 expressed a butyrate receptor GPR109a. Once engaged by butyric acid that concentrated in terminal ileal Peyer’s patches, it was activated and suppressed the cell amplification. In consistent, antibiotic treatment restored the butyric acid caused ILC3 deficit. This process finally benefited effector T cell activation in Peyer’s patches through repressing GM-CSF production from the NCR^+^ ILC3 (Kim *et al*. 2017). In contrast, the ILC3 in colon lamina propria expressed another SCFA-sensitive G protein coupled receptor, Ffar2 (or GPR43). Ffar2 agonists selectively promoted the proliferation and effector function of ILC3. In addition, deletion of *Ffar2* reduced proliferation and IL-22 production of ILC3. These deficits further affected intestinal epithelial cells, leading to reduced expression of mucus-related proteins and antimicrobial peptides, as well as an impaired intestinal epithelial junction (Chun *et al*. 2019). Together, the impact of SCFAs on ILC3s needs to be clarified carefully. The other active microbial metabolites are aromatic hydrocarbons. Aromatic hydrocarbon receptor (AhR) is a critical transcriptional regulator of ILC3. In *Ahr* deficient mice, the number of intestinal ILC3 dramatically reduced, leading to significant defects of CPs and ILFs. The *Ahr* deficient ILC3 also showed reduced expression of c-Kit, IL-7R, as well as anti-apoptotic genes *Bcl2* and *Bcl2l1* that correlated with the deficit (Zelante *et al*. 2013). Several microbial metabolites could work as the AhR agonists. For example, *Lactobacillus* metabolize tryptophan to indole-3-aldehyde to serve as an AhR ligand (Zelante *et al*. 2013). After engaged with its ligand, the AhR translocates from cytoplasm to nucleus, where it pairs with AhR nuclear translocater (ARNT or HIF-1β) and binds to exogenous response elements (XRE) in the genome to regulate the expression of several important downstream genes as just mentioned (McIntosh *et al*. 2010). AhR also enhanced the IL-22 expression, which in turn helped to maintain the diversity of gut microbiota and facilitated the resistance to pathogenic microbes such as *Candida albicans* (Zelante *et al*. 2013). Besides SCFAs and aromatic hydrocarbons, our knowledge about active microbial metabolites is still expanding along with the comprehensive studies of microbiota. In brief, microbiota and particularly the active microbial metabolites are essential for the development, proliferation, and function of ILCs, and thus regulate the gut homeostasis.

#### iNKT

Invariant NKT (iNKT) cell is a distinct component of the immune system and is also crucial in regulating gut homeostasis. It exhibits innate immunity features as indicated by rapid cytokine releasing after stimulation. The iNKT expresses an invariant TCR. The TCR α chain is formed by rearrangement of Vα14 and Jα18 gene fragments, and it pairs with a limited set of Vβ chains. This specific invariant TCR recognizes glycolipid antigens presented by a non-polymorphic MHC class I molecule CD1d (Cianferoni 2013). The development and function of iNKT cells are also affected by microbiota. In mice from different animal facilities, the iNKT cells showed distinct cytokine expression features and a variation of a Vβ7 expressing subpopulation. These phenotypes were attributed to the difference of microbiota composition. In addition, splenic iNKT cells from germ-free mice exhibited an immature phenotype and a decreased reactivity to α-galactosylceramide antigen. However, once exposed to *Sphingomonas* by gavage, the iNKT cell maturation was completely rescued (Wingender *et al*. 2012). In contrast to the splenic iNKT defects, in colon lamina propria and lungs of germ-free mice the mucosal iNKT were found to be accumulated, correlated with a CXCL16 mediated recruitment (Olszak *et al*. 2012). The accumulation of iNKT cells caused a higher morbidity during inflammatory bowel disease (IBD), as well as an increased incidence of allergic asthma. Colonization of conventional microbiota in newborn germ-free mice could efficiently protect the mucosal iNKT accumulation (Olszak *et al*. 2012). Moreover, glycosphingolipids generated from *Bacteries fragilis* could also reduce the iNKT cells in colon, and thus protected the mice from oxazolone-induced colitis (An *et al*. 2014). Therefore, the microbiota and microbial metabolites are necessary to regulate the maturation, proliferation and recruitment of iNKT cells in different tissues.

### Adaptive immunity

Adaptive immune system comprises of T and B cells. The T cell population is further divided into CD4^+^ T helper cells (Th), CD8^+^ cytotoxic T cells, and CD4^+^Foxp3^+^ regulatory T cells (Treg). Immature B cells and naïve T are generated in bone marrow and thymus. Microbiota, and microbial antigens or metabolites, also substantially regulate the development, differentiation and activation of adaptive immune system.

#### Th1 and Th2

CD4^+^ T cells are further divided into different subsets with distinct effector functions. Among them, Th1 and Th2 are critical in maintaining host homeostasis. The impact of microorganisms on Th1 and Th2 balance was described as early as in 1980s in a hygiene hypothesis. In consistent, people also found a tilted Th2 response in germ-free mice, which was reversed by administrating the mice with polysaccharide A (PSA) from *B. fragilis* (van Olst *et al*. 2021). In another study, *Bifidobacterium longum* strain W11 (strain *B. longum* W11) also significantly increased Th1 cytokine production. However, other *B. longum* strains, NCIMB 8809 and BIF53, turned to reduce the Th1 response (Cheng *et al*. 2019a). In opposite, *B. adolescentis* treatment increased Th2 cell number and Th2 responses in colon lamina propria of mice (Kim *et al*. 2021). So, the impact of different microbes on the Th1 and Th2 balance and the underlaying mechanisms still require comprehensive studies. A latest research showed that the bacterial metabolite butyrate benefited the polarization of Th1 through inhibiting histone deacetylase (HDAC) activity (Chen *et al*. 2019). Germ-free mice treated with butyrate also enhanced the expression of Th1 signature genes, T-bet and IFN-γ (Kespohl *et al*. 2017). In brief, the balance of Th1 and Th2 responses is substantially affected by microbiota and microbial-derived metabolites.

#### Th17

Microbiota also affects the differentiation and function of Th17 cells. In germ-free mice, the Th17 cells were absent in intestine (Longman *et al*. 2013). But once colonized with standardized mouse microbiota or a particular SFB bacterium, the Th17 deficiency could be efficiently rescued. The SFB colonization also helped to protect mice from *Citrobacter rodentium* infection, through enhancing the Th17 differentiation (Gensollen *et al*. 2016). The SFB antigen specific CD4^+^ T cells preferentially differentiated towards Th17. Even during a co-infection of SFB and *Listeria monocytogenes*, most SFB-specific T cells were found as Th17, whereas most *L. monocytogenes*-specific T cells were Th1, suggesting that the bacterial antigens were critical in determining the fate of effector Th cells (Longman *et al*. 2013). Colonization of SFB to ileal epithelium stimulated reactive oxygen species (ROS) generation, which promoted IL-1β expression and thus facilitated the Th17 differentiation (Ravindran *et al*. 2016; Tschopp *et al*. 2010). Besides, SFB also induced serum amyloid A 1 and 2 (SAA1/2) in terminal ileum to promote the polarization of Th17 and their IL-17A production (Ravindran *et al*. 2016; Sano *et al*. 2015). Mechanically, the SAA induced IL-23 production from dendritic cells, which was critical for the activation and survival of Th17 (Wingender *et al*. 2012). Other microbes, like *Bifidobacterium*, also affected the Th17 polarization, but the underlying mechanisms still need further studies (Tan *et al*. 2016; Tanabe 2013).

Microbial metabolites also regulate the Th17 differentiation. For example, adenosine 5’-triphosphate (ATP) derived from microbiota stimulated a unique group of CD70^high^CD11c^low^ immune cells in intestinal lamina propria to produce IL-6 and IL-23p19, leading to a promoted Th17 differentiation (Basso *et al*. 2009). Microbial derived AhR ligands also enhanced the differentiation and activation of Th17 (Baricza *et al*. 2016). The impact of another microbial metabolite SCFA on Th17 cells is diverse. While acetate increased Th17 response during *C. rodentium* infection (Cheng *et al*. 2019a), butyrate inhibited the differentiation and function of Th17 by suppressing expression of RORγt, RORα and IL-17 (Chen *et al*. 2019). Another microbial metabolite, PSA from *B. fragilis*, also inhibited the differentiation and function of Th17 through affecting DCs (Jiang *et al*. 2017; Round *et al*. 2011; Round *et al*. 2010). The gut microbiota transforms bile acids into many biologically active molecules which also showed impacts on T cell differentiation. As reported in a latest study, 3-OxoLCA, a derivative of lithocholic acid (LCA), directly inhibited the differentiation of Th17 cells through binding to RORγt (Hang *et al*. 2019). Together, these microbial metabolites are essential in regulating the differentiation and effector function of Th17 cells.

#### Treg

Treg cells play a crucial role in maintaining the host tolerance to commensals. They were also significantly reduced in the colon of germ-free mice. Whereas standardized microbiota or certain colonies of *Clostridium* could efficiently restore the Treg deficiency (Atarashi *et al*. 2011; Sefik *et al*. 2015). *Clostridium* was the most abundant Gram-positive spore bacteria in the gut (Momose *et al.* 2009). Among them, *Clostridium* IV and XIVa were enriched in cecum and proximal colon. There they generated SCFAs and induced gut epithelial cells to secret TGF-β1, both of which could stabilize peripheral Treg and promote their regulatory function (Atarashi *et al*. 2011, 2013).

Microbiota derived metabolites are also involved in regulating the Treg cell differentiation, stabilization and function. Butyrate promoted the differentiation and stabilization of Treg through inhibiting the activity of HDAC and thus increasing histone H3 acetylation at the enhancer region of *Foxp3* (Furusawa *et al*. 2013). PSA from *B. fragilis* promoted Treg differentiation and IL-10 expression through activating TLR2 signal in Treg (Round *et al*. 2010). And, isoalloLCA, a specific bile acid derivate generated by gut commensals, upregulated the expression of Foxp3 and facilitated the differentiation of Treg by inducing mitochondrial reactive oxygen species (mitoROS) (Hang *et al*. 2019). Therefore, gut microbiota and microbial metabolites are required for appropriate differentiation and function of Treg.

#### CD8^+^ T cells

Microbiota and microbial metabolites also regulate cytotoxic CD8^+^ T cells. For example, microbial-derived butyrate could increase the Id2 expression in CD8^+^ T cells, which promoted their antitumor efficiency through an IL-12 signal (He *et al*. 2021). In another instance, bacterial infection increased systemic acetate level in serum. Once uptake by memory CD8^+^ T cells, the acetate promoted glycolysis in the cells and boosted a rapid recall response (Balmer *et al*. 2016).

#### B cells

B cells provide a particularly immune protection through producing antibodies. B cell development in germ-free mice was normal, however, their antibody production was distinct to B cells in SPF mice. In consistent with the hygiene hypothesis, the proportion of IgE^+^ B cells in Peyer’s patches and mesenteric lymph nodes of germ-free mice was increased after weaning. These changes led to an over-reaction to oral administration-induced systemic allergy. Colonizing young germ-free mice with conventional microbes recovered the IgE to normal level, suggesting that intestinal microbiota negatively regulated the IgE production (Cahenzli *et al*. 2013). In addition, the conventional microbiota colonization also restored IgA and IgG1 production in germ-free mice (Hapfelmeier *et al*. 2010). Besides, butyrate also regulated the B cell function. It induced an IL-10-producing B cell population by regulating circadian-clock-related genes (Kim *et al*. 2021).

**Figure 1:**
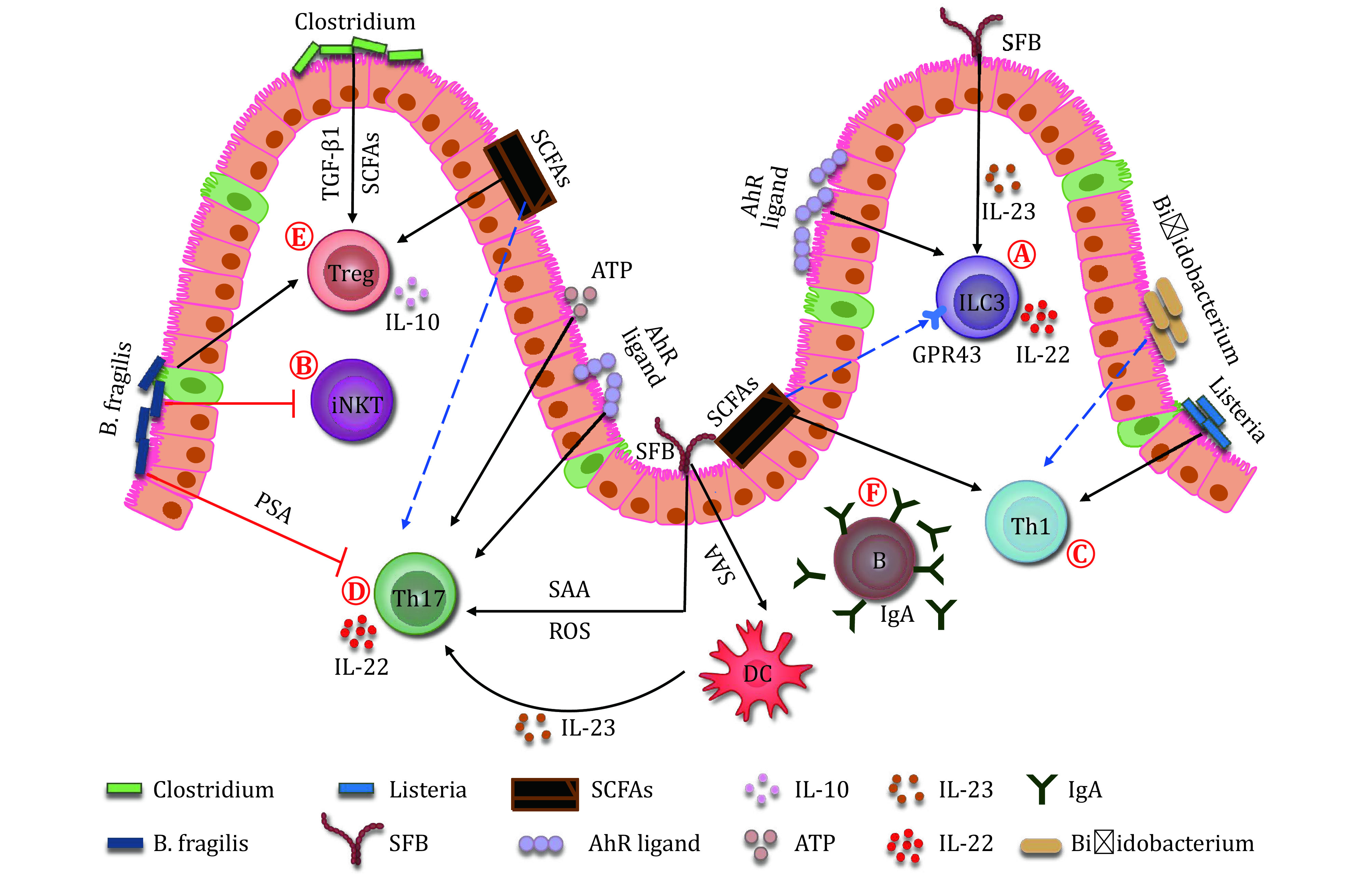
Microbiota helps to shape the immune system. **A** Both microbes and their metabolites regulate ILC3. SFB colonization promotes the production of IL-22 from ILC3 in an IL-23-dependent manner. Butyric acid negatively regulates the NCR^+^ ILC3. Whereas, SCFAs selectively promote the proliferation and effector function of colonic ILC3 through binding to GPR43. The Ahr ligand generated from microbes also regulates ILC3 number and their IL-22 production. **B** *B. fragilis* reduces the number of iNKT cells in the gut. **C** Microbes and their metabolites affect Th1 response. *Bifidobacterium* influences the Th1 cytokine production. *L. monocytogenes* induces the Th1 differentiation. Also, a microbial metabolite, butyrate, benefits the Th1 polarization. **D** Microbes and their metabolites regulate the differentiation and effector function of Th17. SFB facilitates the differentiation of Th17 through promoting ROS and SAA production from the ileal epithelial cells. The SAA also benefits the activation and survival of Th17 by stimulating IL-23 production from dendritic cells. The ATP derived from microbiota also stimulates the differentiation of Th17 cells. SCFAs, such as butyrate, inhibits the polarization of Th17 cells, however, acetate promotes the Th17 responses. *B. fragilis* produced PSA inhibits the differentiation of Th17. The AhR ligands enhance the IL-22 secretion from Th17. **E** Microbes and their metabolites regulate Treg differentiation and function. *Clostridium* generates SCFAs, and induces the epithelial cells to produce TGF-β1. Both SCFAs and TGF-β1 can stabilize peripheral Treg and promote their function. The PSA produced by *B. fragilis* promotes the differentiation of Treg cells and their IL-10 expression. The butyrate promotes the differentiation of Treg. **F** Conventional microbiota deletion reduces the IgA secretion from B cells

In summary, the development and function of innate and adaptive immune cells are broadly regulated by microbiota. Mechanically, microbial-derived antigens or particular metabolites are found to affect the antigen presentation, signaling transduction, or transcriptional regulation in innate or adaptive immune cells (Fig. 1). Hence, a proper colonization of microbiome after birth is necessary for the normal development and education of host immunity.

## HOST-MICROBIOTA INTERACTIONS IN DISEASES

In opposite to the fundamental role in promoting host immunity and tissue homeostasis, the microbiota also associates with many diseases through disturbing the protective function of immune system. In the following part, we will discuss how microbiota and microbial metabolites influence the susceptibility of host to various diseases, such as infectious diseases, autoimmune diseases, and metabolic diseases.

### Infectious diseases

Gastrointestinal infection is a global health concern. A major human diarrheal pathogen is *Vibrio cholerae*, which affects millions of people annually (Clemens *et al*. 2017). A previous study demonstrated that a few innate immunity-associated molecules, including NF-κB, MAPK and TLRs, were activated during early *V. cholerae* infection (Bourque *et al*. 2018). However, the intestinal disruption would still last for over a month, which might protect the host from a secondary infection by *V*. *cholera* or other pathogens. Metagenomic studies revealed an obvious difference between the microbiota in *V*. *cholera* infected patients and that in healthy people (Alavi *et al*. 2020). Further studies indicated that *Blautia obeum* abundance significantly correlated with *V. cholerae* resistance (Alavi *et al*. 2020). Together, the protection against *V. cholerae* infection was provided by both a long lasting immune response and the abundance of particular commensals such as *B. obeum*. *Helicobacter pylori* is another pathogen commonly associated with the initiation and progression of peptic ulcers and stomach cancer (Pucułek *et al*. 2018). In a latest research, *H. pylori* infection was found to induce a quick stomach infiltration of ILC2 (Satoh-Takayama *et al*. 2020). IL-5 produced by ILC2 promoted IgA expression from B cells, which then coated on the *H. pylori* and helped with its clearance. Taking advantage of culture-independent next-generation sequencing (NGS) technology, we are now able to gain more knowledge about the microbial communities (metagenomics) and their involvements in the incidence or progression of infectious diseases (Tay *et al*. 2016). However, further studies about the underlying mechanisms of microbe–microbe or microbe–host interactions are still needed to get a comprehensive understanding of these infectious diseases.

### Autoimmune diseases

Autoimmune diseases, such as multiple sclerosis (MS), rheumatoid arthritis (RA), and inflammatory bowel disease (IBD), affect approximately 10% of the world population (Gawalko *et al*. 2020). MS is the most common demyelinating disorder. There were about 1.6 to 1.95 million MS patients in 2017 in the world (Brownlee *et al*. 2017). Microbiota is found to correlate with MS incidence. In a study of spontaneous brain autoimmunity with transgenic mice model, microbiota from MS patient induced a significantly higher incidence of brain autoimmunity in the mice than that from his/her healthy-twin. In consistence, IL-10 production from the immune cells of the mice receiving the MS-twin microbiota was significantly reduced (Berer *et al*. 2017). Microbial metabolites, such as PSA derived from *B. fragilis*, was able to induce the differentiation of CD4^+^ T cells to IL-10 producing Tregs (Round *et al*. 2010, 2011). In addition, mice colonized with *B. fragilis* showed a significant recovery from EAE, another mouse model of MS (Ochoa-Repáraz *et al*. 2010a). Also, oral administration of PSA protected EAE through a TLR-2-dependent manner (Ochoa-Repáraz *et al*. 2010b; Wang *et al*. 2014). Thus, the application of *B. fragilis* or PSA may provide a promise for the MS patients in future. SCFAs are also involved in the MS occurrence. The abundance of SCFA-producing bacteria in the MS patients was decreased. And, propionic acid (PA) in their serum and feces was also reduced. These reductions caused an imbalance between Treg and Th17, and increased the severity of the disease. PA supplementation to EAE mice enhanced their Treg function, and thus delayed the disease progression (Duscha *et al*. 2020). The microbiota–host interaction in human, however, is sometimes distinct with that in mice. For example, in mice the *B. fragilis* had an obvious anti-inflammatory function, but in human it led to inflammation. The impact of microbiota on autoimmune patients was also influenced by their genetic backgrounds, dietary habits and lifestyles (Yurkovetskiy *et al*. 2015). Thus the precise mechanisms by which microbiota affects the pathology of autoimmunity in human patients still need further investigation.

### Metabolic diseases

Metabolic diseases such as type 2 diabetes, cardiovascular diseases, and fatty liver diseases are frequently concurrent with obesity. There are over 1.9 billion obese people in the world. The occurrence of metabolic diseases in obese people is frequently correlated with their microbiota dysbiosis (Han *et al*. 2014; Tang *et al*. 2017; Wang *et al*. 2011) and chronic inflammation (Saltiel *et al*. 2017). During obesity, gut microbiota alteration is usually observed, including changes of specific microbial populations and reduction of microbial gene richness (MGR) (Muscogiuri *et al*. 2019). One important evidence correlating the microbiota alteration to obesity was that fecal microbiota from obese donor mice was sufficient to cause obesity in recipient mice.

Microbial-derived metabolites also contribute to obesity as well as metabolic diseases. The gut barrier consists of physical, biological and immunological components, normally protects pathogenic microbial metabolites from the host (Wells *et al*. 2017). For example, goblets, a type of specialized gut epithelial cells, secret mucus and particular glycoprotein mucins to form a mucus layer against enteric bacteria or pathogen invasion (Derrien *et al*. 2010). Thus, microbial metabolites such as lipopolysaccharide (LPS) can hardly enter the circulation through gut barrier in normal conditions. Ever if there is very few LPS leakage, it would be quickly cleaned by immune system or degraded in liver via detoxification (Huang *et al*. 2016). However, obesity or diabetes will lead to a reduced mucus thickness in mice (Everard *et al*. 2013; Li *et al*. 2016) and human (Chassaing *et al*. 2017), which correlated inversely with body mass index (BMI), blood glucose levels and glycosylated hemoglobin. The thin mucus layer increased the leakage of LPS to circulation, resulting to augmented plasma LPS. In the situation of obesity, plasma LPS level was at least doubled (Lassenius *et al*. 2011). The increased plasma LPS led to a metabolic endotoxemia and a systemic inflammation (Fuke *et al*. 2019), which finally caused insulin resistance and various metabolic diseases. Therefore, the microbiota and its impact on the immune system should be considered in future for various metabolic diseases (Sittipo *et al*. 2018).

### Neurological diseases

Gut microbiota also plays a critical role during neuron system development. Microglia cells in brain perform a canonical myeloid cell function, including phagocytosis and initiating pro-inflammatory responses (Nayak *et al*. 2014). It was found that germ-free mice were less resistant to LPS stimulation and LCMV infection (Matcovitch-Natan *et al*. 2016). Transcriptome analysis suggested that the microglia from the mice exhibited an immature phenotype that caused impaired immune responses. Microbial recolonization partially restored the defect, indicating that microbiota was involved in regulating the maturation and function of microglia (Erny *et al*. 2015). Also, microbiota was found involved in the incidence and recovery of CNS injury. In a mouse model of middle cerebral artery occlusion (MCAO)-induced ischemic brain injury, intestinal bacteria promoted infiltration of IL-17^+^ γδT cells and their responses in the brain, resulting to a CNS injury. However, once the mice were treated with amoxicillin and clavulanic acid, they showed a reduced infarct volume and an improved sensorimotor function. This neuroprotective effect was related to decrease IL-17^+^ γδT cell infiltration in meninges (Benakis *et al*. 2016). Nonetheless, other antibiotics, such as ciprofloxacin and metronidazole, turned to reduce the mice survival following MCAO, indicating that different microbial species had distinct impacts on the brain injury (Winek *et al*. 2016). Therefore, both microbiota and immune system were implicated in neurologic pathologies (Dinan *et al*. 2017), however, further studies about the underlying mechanisms are still needed to clarify how particular microbes and specific immune cells cooperate to regulate CNS function.

**Figure 2:**
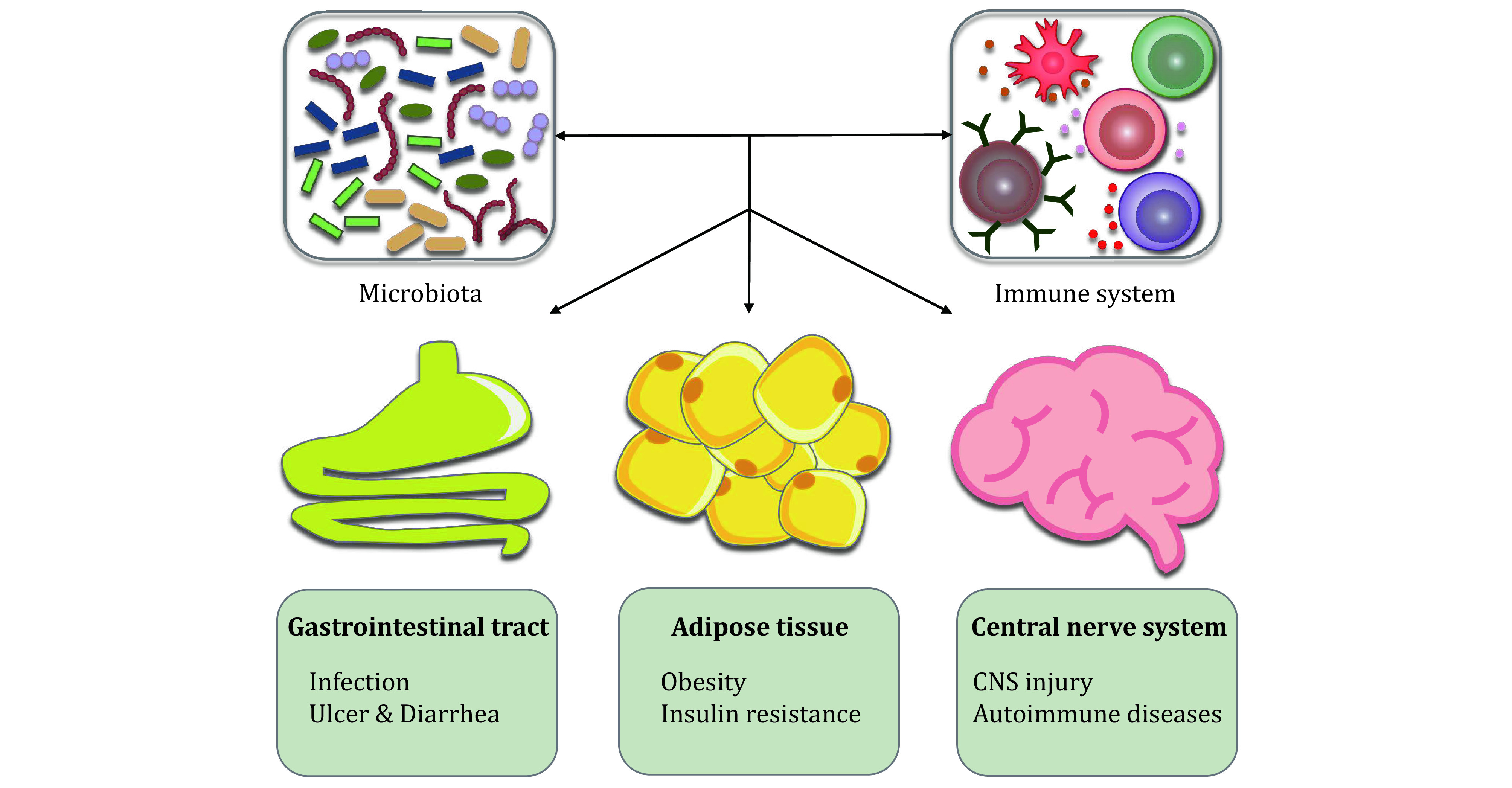
The host-microbiota interaction in diseases. Microbiota and their metabolites regulate the development and function of the immune system, and thus influence diseases in multiple tissues. In the gastrointestinal tract, the microbiota dysbiosis causes infectious diseases, and the host shows symptoms of ulcer and diarrhea. In the adipose tissue, the microbiota contributes to metabolic diseases, such as obesity and insulin resistance. In the central nerve system, the microbiota involves in the incidence and recovery of CNS injury and autoimmune diseases

In brief, microbial dysbiosis correlates with many diseases (Fig. 2). Gut microbiota is frequently found to be disturbed in infectious diseases such as *H. pylori* induced peptic ulcers. Microbiota also contributes to the occurrence of autoimmune diseases, including multiple sclerosis, rheumatoid arthritis, and inflammatory bowel disease. Moreover, as a cause of chronic inflammation, the microbiota is involved in the incidence of metabolic diseases and neurological diseases. Manipulating the microbiota alteration in these diseases may serve as a potential therapeutic strategy in future.

## IMMUNE SYSTEM REGULATES MICROBIOTA COMPOSITION AND GUT HOMEOSTASIS

While microbiota helps to shape the host immune system, the host immunity also regulates microbiota composition and gut homeostasis in reverse. Gastrointestinal tract includes a mucus layer, an epithelial layer, and a lamina propria layer. The mucus and epithelial layers serve as a chemical barrier and a physical barrier, which protect the gut flora in the lumen from the host cells. Whereas, the lamina propria that contains numerous immune cells such as T cells, B cells, ILCs, macrophages, DCs, and intraepithelial lymphocytes (IELs), mainly plays an immune barrier function (Allaire *et al*. 2018).

The immune barrier is critical in regulating gut microbiota composition and maintaining gut homeostasis. For example, IL-22 produced by ILC3 and Th17 cells induced gut epithelial cells to generate essential regulators for microbiota, including antimicrobial peptides (β-defensins, RegⅢβ, RegⅢγ), calcineurin (S100A8, S100A9) and lipoprotein 2 (Parks *et al*. 2015). Antimicrobial peptides directly killed or inhibited the growth of microbes. Calprotectin formed by S100A8 and S100A9 heterodimer could sequester zinc and manganese, preventing microbes from these nutrients. And, lipocalin-2 bound to siderophore enterochelin to limit iron availability in the gut (Cheng *et al*. 2019b). IL-22 also induced epithelial cells to secrete high levels of mucus-related molecules, including Muc1, Muc3, Muc10, and Muc13, which prevented gut microorganisms from transmitting across the epithelial barrier during steady state and inflammation (Sonnenberg *et al*. 2011). Furthermore, IL-22 could upregulate α1,2-Fucosyltransferase-2 (Fut2) expression in gut epithelial cells, which eventually increased fucosylation of gut epithelium. Once shedded to gut lumen, the fucose residue could serve as dietary carbohydrates for commensals, and thus competitively promoted microbial balance (Pickard *et al*. 2014). Besides IL-22, other cytokines, like IL-17F, produced by Th17 and ILC3 also promoted the production of antimicrobial peptides in the epithelial cells (Domingues *et al*. 2020).

Th and ILC activation induce tissue inflammation to suppress pathogenic microbes in the gut. IELs, mainly including αβ^+^ and γδ^+^ T cells, locate between epithelial cells. They played a critical role in protecting epithelial damage and preventing microbial transmission through producing inflammatory cytokines, such as IFN-γ and keratinocyte growth factors (KGF) (Olivares-Villagomez *et al*. 2018). Treg cells are the other crucial regulators in the gut which help to suppress inflammation and maintain immune tolerance. IL-10 produced by Treg has a critical immune suppressive function. The IL-10 and Treg were indispensable for repressing pro-inflammatory T cells in mice infected by *H. hepaticus*, and thus protect epithelial damage (Takeshi Tanoue 2010).

Besides T cells and ILCs, other immune cells also involved in regulating the microbiota composition and gut homeostasis. DCs and macrophages are responsible for the identification and clearance of pathogenic bacteria. DCs could protrude their synapses through intestinal epithelium to the gut lumen to sense pathogenic microbes such as *Salmonella* and *E. coli*, and then initiated both innate and adaptive immune responses against the pathogens (Liu *et al*. 2018). Secretory IgA (sIgA) produced by B cell also had a crucial role in regulating microbiota composition through coating on colitogenic bacteria and promoting their clearance (Palm *et al*. 2014).

**Figure 3:**
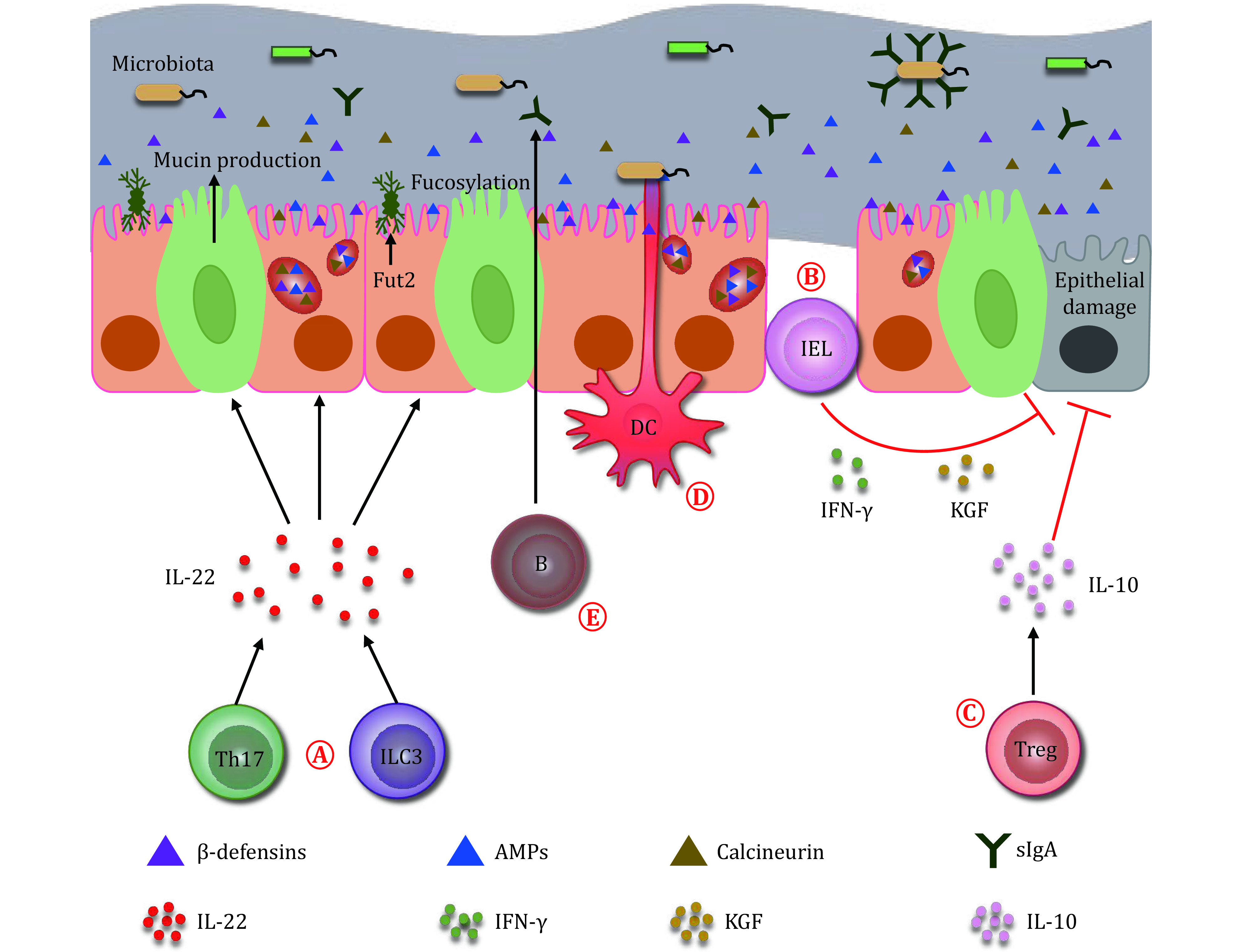
Immune system regulates the microbiota. **A** Microbiota is regulated by ILC3 and Th17 cells. The ILC3 and Th17 cells inhibit the growth of microbiota through producing IL-22 and inducing epithelial cells to secrete β-defensins, antimicrobial peptides and calcineurin. The IL-22 also induces epithelial cells to secrete mucin, and thus represses the transmission of gut bacteria. In addition, the IL-22 upregulates Fut2 expression in the epithelial cells, and increases the fucosylation on their surface, which benefits the proliferation of commensals in the gut. **B** IELs protect the damage of epithelial cells through producing cytokines such as IFN-γ and keratinocyte growth factors (KGF). **C** Treg cells repress the damage of epithelium by secreting IL-10. **D** DCs protrude their synapses through the intestinal epithelium to the gut lumen to sense the microbiota. **E** B cells produce secretory IgA (sIgA) to the gut lumen. The sIgA coats on the colitogenic bacteria to help with their clearance

In summary, immune cells are indispensable in regulating the microbiota composition and gut homeostasis (Fig. 3). DCs directly sense the microbiota in gut lumen and transduce the microbial signals to other immune cells, like ILC3 and Th17. They then produced IL-22 to regulate epithelial cells to secret microbial regulators, preventing pathogenic bacteria from invasion. B cells produced sIgA also helps to clear colitogenic bacteria. Meanwhile, IELs and Treg protect the epithelium from damages. In brief, these immune cells, epithelial cells, microbiota interplays suppress pathogenic bacteria from adhering to the surface of the intestinal mucosa, help to balance the composition of microbiota, and protect the host from infectious or inflammatory diseases.

## CONCLUSIONS AND PERSPECTIVES

As discussed above, microbiota and immune system mutually interact with each other. The microbiota shapes the development and function of both innate and adaptive immune systems. But in various disease conditions, it also affects protective immune functions leading to disease occurrence or progression. In reverse, the immune system also regulates the balance of microbiota, keeping the host tolerance to commensals but promoting the clearance of pathogenic microbes. This microbiota-immune system interplay is critical in maintaining host homeostasis. For future application, fecal microbial transplantation seems to be a promising therapeutic strategy for certain patients. However, our knowledge about the precise mechanisms underlying the communication between microbiota and local or systemic immunity is still quite limited currently. Further investigations correlating the microbiota alterations in health and diseases with host immunity changes are required to promote the clinical application of microbiota. The microbial community also consists of viruses, fungi, and protozoan. Their interplay with the host immunity and roles in health and diseases are still quite elusive, and thus are not involved in this review. But certain recent studies indicate that they should never be omitted. Furthermore, integration of ecological, genomic, microbiological and immunological approaches is also required in the future microbiota studies.

While increased evidences link most human diseases with gut microbiota dysbiosis, whether this disorder is a cause or a consequence is still largely unclear. Human microbiota-associated rodent models are frequently used to study the relationship between the alerted microbiota and the occurrence of relative diseases. However, it should also be aware that numerous human gut microbes were unable to colonize in recipient animals (Zhang *et al*. 2017). Also, genetic alteration, dietary habits, and lifestyles of those patients with gut dysbiosis may be critical for the disease phenotypes, but they are usually difficult to be recapitulated in experimental animals (Arrieta *et al*. 2016). Thus, more suitable tools for microbial and immunological investigations are still needed in the future.

## Conflict of interest

Di Wu, Yinlian Zhang, Suwei Dong and Chao Zhong declare that they have no conflict of interest.
